# 

**Published:** 1912-09-28

**Authors:** 


### Gifts and Bequests.

Mr. Henry Hugh Wippell, of Exeter, has bequeathed £500 to the Royal Devon and Exeter Hospital.

Miss Emmeline Ellen Hickman, of Salisbury, has bequeathed £200 to the Nottingham General Hospital.

The Chesterfield and North Derbyshire Hospital has received a legacy of £22 12s. from the executors of the late Miss M. H. Bennett.

The Chelsea Football and Athletic Company, Limited, have given £20 to the Royal Hospital for Incurables, Putney Heath, being part proceeds of practice matches.

Mr. Alfred Rees Lewis, of Camden Road, N.W., has bequeathed £500 to the Royal Free Hospital, Gray's Inn Road; £300 each to the Hospital for Women, Soho Square, the British Lying-in Hospital, the London Temperance Hospital; and £200 each to the National Hospital for Diseases of the Heart, Soho Square, the Westminster General Dispensary, and to the Surgical Aid Society.

The late Mr. Herbert James Garrod, of Cheveley, Newmarket, for many years an annual subscriber to Addenbrooke's Hospital, Cambridge, has bequeathed to that institution the sum of £200. One of his executors will be appointed an honorary governor for life in pursuance of bye-law 49. The late Mr. Henry J. King, of Wrestlingworth, Beds, has bequeathed £500, less duty, to the hospital, of which institution he was a life governor and an annual subscriber for many years. A cot will be named after the benefactor, and a brass tablet erected over the cot. One of the executors will also be appointed an honorary governor for life. Two hundred pounds has been received from the trustees of the Elizabeth Butterfield Fund, per W. R. Wherry, J.P., and George Wherry, M.C., F.R.C.S. One of the trustees will be appointed an honorary life governor. A special donation of £100 has also been received from C. Finch Foster, J.P., of Cambridge.

### Appointments.

Mr. Lennox Lee, of Kersal, has been appointed a member of the board of management of Salford Royal Hospital, to fill the vacancy caused by the resignation of Mr. Astley Jepson.

Dr. Robert P. M'Donnell, of Dublin, has been appointed tuberculosis medical officer in charge of the new dispensary to the Roscommon County Council under the Irish National Insurance Act.

Mr. J. B. Ferguson Wilson, F.R.C.S. (Eng.), and Mr. Vincent Townrow, F.R.C.S. (Eng.), have been elected honorary assistant surgeons to the Sheffield Royal Hospital for the ensuing seven years.

Dr. W. J. Young, of the Lister Institute, has been appointed biological chemist to the Australian Institute of Tropical Medicine. He graduated at Victoria University, and is a D.Sc. of London.

Dr. Thomas Roberts has been appointed county medical officer of health to the Denbighshire County Council. Dr. Roberts, who has held a similar position to the Wrexham Urban and Rural District Councils, graduated M.B., C.M. at Edinburgh in 1895, and holds the Diploma of Public Health of Liverpool.

The following appointments have been made from October 1 for the ensuing six months at the Salford Royal Hospital :—To be resident surgical officer, Dr. R. W. Duncan, L.R.C.P. and S. (Edin.), L.F.P.S. (Glas.); house physician, Dr. James A. Tomb, M.B., Ch.B. (Edin.); house surgeon, Dr. Caleb Davies, M.B., Ch.B. (Vict.); junior house surgeon, Dr. Ronald B. Berry, M.B., Ch.B. (Vict.); casualty house surgeon, Dr. Thomas H. Houston, M.B., Ch.B., B.A.O. (Q.U.I.).

### BUREAU OF INFORMATION.

### Work Among British Patients Abroad.

The establishment of The Hospital Bureau of Information has exposed the very general desire on the part of medical men, nurses, and the general public to obtain full and accurate data relating to facilities for nursing English-speaking patients in foreign countries. These facilities vary greatly from one place to another. In many localities on the Continent, for instance, British nurses are still unknown. In others they are supplied at great cost from the staff of London hospitals as required. In others, again, there are agencies supplying British nurses, pay hospitals where they are employed, and medical men, of various nationalities, who are thankful to get them whenever they are to be had. These varying conditions prevail not only in foreign countries, but also in British Colonies. It is often of the utmost importance to persons travelling to distant places to ascertain before they start what opportunity offers in case of illness for being nursed by women of their own speech and with familiar methods. Moreover, nurses who desire to take posts abroad are frequently at a loss to know something about the conditions awaiting them. They seek a centre of information and advice, and none is available.

With these facts in view we are instituting an extensive inquiry into the extent to which British trained nurses are needed or actually employed in places frequented by English-speaking people. This inquiry is now in progress. As returns come in we shall be in a position to place our readers in possession of many valuable details relating to nursing homes or "cliniques" abroad, the spread of the British trained nurse throughout the civilised world, and the opportunities which offer for increasing the numbers of those employed outside these islands.

### Rules for Correspondents.

1. Every letter, on whatever subject, **must be accompanied by the coupon** to be cut from the back cover (inside page) of The Hospital, current issue, and must contain the name and address of the correspondent with pseudonym for publication if desired.
2. Replies by post cannot be given, save under exceptional circumstances at the Editor's discretion.
3. Letters from Approved Homes in reply to special needs published in the Bureau should state terms and full particulars, and be sent prepaid under cover to the Editor of the Bureau with coupon and name enclosed for identification.
4. Proprietors of Homes which have not yet been entered on the List of Approved Homes, but have spare accommodation likely to suit special needs, are invited to write for an application form for registration. The fee for registration, which includes two announcements of the Home in the Bureau, is 5s.
5. Special needs of every description relating to those who are sick or in difficulties are made known in these columns **free of charge.**
6. All communications to be addressed to The Editor of The Hospital, 28 Southampton Street, Strand, London, W.C., and marked "Bureau of Information."

### Homes Wanted.

**For Slight Mental Case.**—The patient is a lady of fifty-one, a widow, suffering from partial loss of memory and occasional severe headache; not helpless, but requiring firm and kindly control and companionship in walks. The relatives can only afford a small sum. Applications should state the lowest terms, and be sent under cover of the Bureau to Mac. (122a).

**For Alcoholic Patient.**—Middle-aged lady suffering from chronic alcoholism, whose friends cannot afford to pay more than £2 2s. a week. We are glad to learn that you have been able to select a suitable home from the names forwarded to you from the Bureau, and that the patient has already been received in one of our Approved Homes. In cases of emergency we are always pleased to adopt this course, and forward addresses direct to medical men.—Dr. R. (123a).

### Approved Homes.

Note.—*No Home is added to the List without direct medical and other testimony to its good management.*

**Peterborough.**—Rothesay Villas, 73 Lincoln Road. Proprietress: Miss Warburton. Medical, surgical, maternity cases. Special experience with Weir-Mitchell treatment. Chronic patient can be taken at low terms. Fully-trained nurses supplied from 31s. 6d. weekly.

**High-Class Nursing Home in London.**—28 Cleveland Gardens, Hyde Park, W. Proprietress: Miss L. M. Patten. Maternity, heart, rest-cure, chronic cases. All branches of massage; Swedish exercises; Nauheim treatment. Close to Kensington Gardens. Family life, if desired. Specially adapted for children.

### Needs and Helpers.

**Rescue Home for Children.**—The child you speak of could be received at the new home for ruined children at St. Mary's, Buxted. Write to the Mother Superior. Children are received between the ages of six and twelve, and are fed, clothed, and educated at an inclusive charge of 8s. 6d. per week. The home is in beautiful and healthy surroundings, and under highly-trained and sympathetic management. Very encouraging results have been obtained in the difficult task of restoring the little inmates to innocence.

### Training and Employment.

**Light Permanent Post.**—We should be very happy to help you if we could, and it is possible that one of our readers may be able to put you in the way of procuring the work you are seeking. We note that you are forty-five years of age, that you are a trained nurse with twenty years' experience, and that you would like to assist the matron in some small home or hospital for a low salary, or to take charge. Write to Sister Smith, 15 Buckingham Street, Strand, W.C. She is often asked to fill vacancies of this nature.—E. L., Leominster.

**Bureau for Nurse Attendants.**—See reply to E. L. above. We know of no other bureau for the employment of chronic nurses, companions, etc. But, if you are a member of the Pension Fund, Miss Rosa Smith will be able to help you, and if you are not, well, it is not a disability which lacks a remedy.

**Inexpensive Training for Nursery Nurses.**—The expense is, as you say, a great bar to taking the ordinary course at training schools for this branch of work. You will be glad to know, however, that the Women's Industrial Council have now founded a training home for this purpose at 4 King Edward Road, Hackney, N.E., where the fees are very moderate—viz. £30 a year.—Ruby.

**District Nurse Wanted.**—We fear we must refer you to our advertising columns, or those of *The Nursing Mirror*, for this "want." We are unable to undertake recommendations of this nature in the Bureau.—Longworth.

